# Exploring the Immune Infiltration Landscape and M2 Macrophage-Related Biomarkers of Proliferative Diabetic Retinopathy

**DOI:** 10.3389/fendo.2022.841813

**Published:** 2022-05-27

**Authors:** Zhishang Meng, Yanzhu Chen, Wenyi Wu, Bin Yan, Yongan Meng, Youling Liang, Xiaoxi Yao, Jing Luo

**Affiliations:** ^1^Department of Ophthalmology, The Second Xiangya Hospital, Central South University, Changsha, China; ^2^Department of Radiation Oncology, Hunan Cancer Hospital and The Affiliated Cancer Hospital of Xiangya School of Medicine, Central South University, Changsha, China; ^3^Department of Ophthalmology, Xiangya Hospital, Central South University, Changsha, China; ^4^Shenzhen College of International Education, Shenzhen, China

**Keywords:** proliferative diabetic retinopathy, biomarkers, M2 macrophage, immune landscape, bioinformatics

## Abstract

**Backgrounds:**

Diabetic retinopathy (DR), especially proliferative diabetic retinopathy (PDR), is the major cause of irreversible blindness in the working-age population. Increasing evidence indicates that immune cells and the inflammatory microenvironment play an important role during PDR development. Herein, we aim to explore the immune landscape of PDR and then identify potential biomarkers correlated with specific infiltrating immune cells.

**Methods:**

We mined and re-analyzed PDR-related datasets from the Gene Expression Omnibus (GEO) database. Using the cell-type identification by estimating relative subsets of RNA transcripts (CIBERSORT) algorithm, we investigated the infiltration of 22 types of immune cells in all selected samples; analyses of differences and correlations between infiltrating cells were used to reveal the immune landscape of PDR. Thereafter, weighted gene co-expression network analysis (WGCNA) and differential expression analysis were applied to identify the hub genes on M2 macrophages that may affect PDR progression.

**Results:**

Significant differences were found between infiltration levels of immune cells in fibrovascular membranes (FVMs) from PDR and normal retinas. The percentages of follicular helper T cells, M1 macrophages, and M2 macrophages were increased significantly in FVMs. Integrative analysis combining the differential expression and co-expression revealed the M2 macrophage-related hub genes in PDR. Among these, *COL5A2, CALD1, COL6A3, CORO1C*, and *CALU* showed increased expression in FVM and may be potential biomarkers for PDR.

**Conclusions:**

Our findings provide novel insights into the immune mechanisms involved in PDR. *COL5A2, CALD1, COL6A3, CORO1C*, and *CALU* are M2 macrophage-related biomarkers, further study of these genes could inform novel ideas and basis for the understanding of disease progression and targeted treatment of PDR.

## Introduction

Diabetic retinopathy (DR) is a major cause of irreversible blindness in the working-age population worldwide (1, 2). Proliferative diabetic retinopathy (PDR), characterized by neovascularization (NV) and excessive proliferation, is the most advanced and refractory form of DR (3). The global incidence of PDR is 1.4% among all individuals with diabetes (4). PDR management has been one of the greatest challenges in modern ophthalmology. Recent advances in high-throughput technologies and bioinformatics analysis have improved the understanding of PDR and facilitated the development of treatment (5). Therefore, the identification of new biomarker and therapeutic strategies for retinal NV in PDR is urgent and in high demand for improved clinical outcome.

Retinal cells are privileged from systemic immune surveillance (6). Once the blood-retina barrier is breached, retinal cells gate the layer of protection by suppressing the local inflammatory response (7). Immunological activity in the central nervous system (CNS) is largely determined by an innate immune response and is heightened in PDR (8). Previous studies have shown enrichment of immune processes in DR (9, 10). In addition, therapies targeting inflammation have also been shown to be effective for patients with DR (11, 12). However, whether and how molecular pathways and immune cell populations are involved in the progress of PDR is still unknown.

Activated macrophages are generally divided into M1-like and M2-like forms (13). The activation of M2 macrophages plays a major role in cellular processes such as cell proliferation and differentiation. These processes rely on external factors including transcription factors, intrinsic-signaling pathways, cytokines, and metabolic adaptation (14). Previous studies have demonstrated the key signals involved in M2 macrophage polarization in DR (10, 15, 16). A recent study also showed that M2 Macrophages are the major immune cells that involved in the immune response of retinal neovascularization in the rat model with ischemic retinopathy (17).

In the present study, we analyzed the transcriptome data from publicly available databases using various bioinformatics algorithms to identify potential immune cell-related biomarkers in PDR and a panel of M2 macrophage-related biomarkers, and to determine the immune landscape of PDR.

## Materials and Methods

### Data Collection and Preprocessing

The gene expression data and relevant clinical information were downloaded from the Gene Expression Omnibus (GEO) database (http://www.ncbi.nlm.nih.gov/geo/) with accession numbers GSE60436 (18) and GSE102485 (19). Fibrovascular membranes (FVMs) obtained from PDR patients and normal retinal tissue from donated eyes in the above two datasets were included in this study. The expression data were then normalized using the “normalizeBetweenArrays” function in the “limma” R package (20), and the “sva” R package was used to correct for batch effects between two different arrays (21).

### Gene Set Enrichment Analysis

GSEA was performed between FVMs and normal retinas to explore the potential biological functions involved in the occurrence and development of PDR; the “Hallmark” gene sets (from the Molecular Signatures Database) were selected as the annotation gene sets (22). Significance was set at NOM *p*<0.05 and false discovery rate (FDR)-adjusted *q*‐values <0.25.

### Immune Cell Infiltration-Related Analysis

The CIBERSORT algorithm was applied to estimate the relative proportions of 22 types of immune cells which infiltrated into all included samples (23), those with a CIBERSORT output of *p*<0.05 were considered accurate and enrolled for further construction of the immune landscape, otherwise, samples were eliminated. Thereafter, the correlation matrix of different immune cells was constructed and visualized using the “Corrplot” R package. Principal component analysis (PCA) was then used to evaluate subsets distinguished by the extent of immune cell infiltration. The results were visualized using the R package “scatterplot3d.”

### Construction of Co-Expression Network Associated With PDR-Infiltrating Immune Cells

To search for genes associated with immune infiltrates, we constructed a signed hybrid co-expression network for all selected samples using weighted gene correlation network analysis (WGCNA) (Soft-power 5, mergeCutheight 0.25, minModuleSize 20) (24). After filtering out low-expression genes to remove noise, those genes with similar expression patterns were hierarchically clustered into several modules according to the topological overlap measure-based dissimilarity, and all the modules were summarized by module eigengenes (ME) which were used to measure the module membership. The hub genes in our study were defined as those with ME-based gene connectivity (kME) >0.9 within each module; those genes with higher kME tended to have higher connectivity. We then performed correlation analysis between co-expression modules and immune infiltrated cells in PDR to identify hub genes associated with the disease. Pearson’s correlations were calculated between the ME and immune cells obtained from previous analysis. Associations between the module and immune cell were considered significant at *p*<0.05. Thus, we obtained the module and hub genes that likely shaped the specific immune landscape of PDR. Meanwhile, analysis of gene ontology (GO) annotation and enrichment was carried out to better understand the biological functions of these enrolled genes (R packages “clusterProfiler” and “enrichplot,” *p*<0.05, *q*<0.05).

### Identification Biomarkers of PDR-Infiltrating Immune Cells

In general, clues about the disease are inferred from gene expression differences between normal and pathologic tissues. Therefore, we conducted differential expression analysis of FVMs and normal retinas. The differentially expressed genes (DEGs) were identified using the “limma” R package and filtered by |log2FoldChange| >1 and FDR<0.05. The potential biomarkers were screened by integrating the results of the differentially expressed analysis, WGCNA, and immune landscape of PDR. The expression distribution of selected genes was visualized with violin plots using R packages “ggplot2” and “reshape2.” Finally, PCA was used to evaluate the discriminative capacity of these candidates to differentiate the two groups.

## Results

### Overview of Research Design and Data Preparation

**Figure 1:**
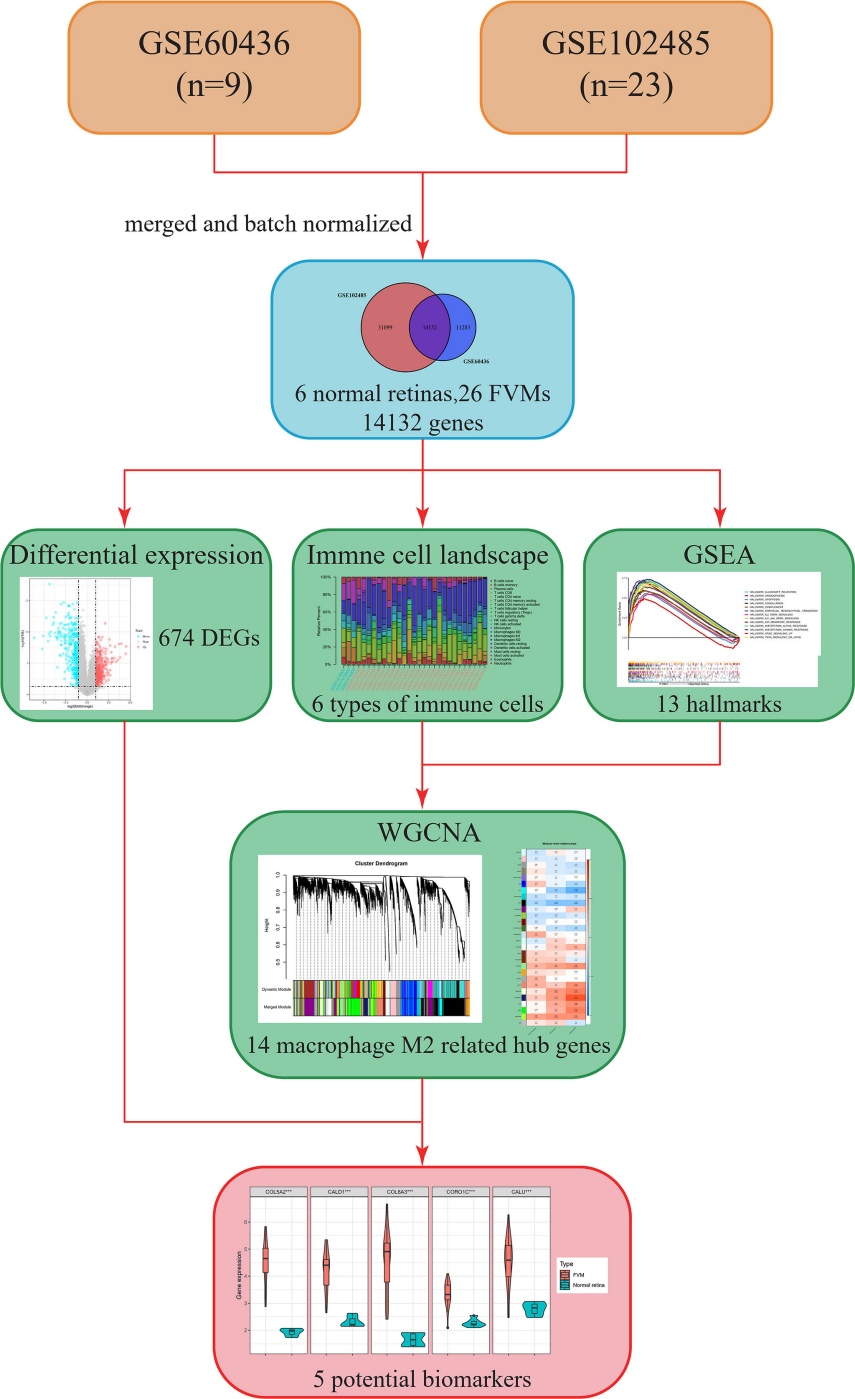
Flow chart showing the research process in this study. FVM, fibrovascular membrane; DEG, differentially expressed gene; CIBERSORT, cell-type identification by estimating relative subsets of RNA transcripts; GSEA, gene set enrichment analysis; WGCNA, weighted gene co-expression network analysis.

The overall design and workflow of this study are shown in **Figure 1**. An integrative analysis combining differential expression and co-expression revealed that immune cells, particularly M2 macrophages, play an essential role in the progression of DR, and further suggested that increased expression of *COL5A2, CALD1, COL6A3, CORO1C*, and *CALU* in the FVM may be potential M2 macrophage-related biomarkers for PDR.

**Figure 2:**
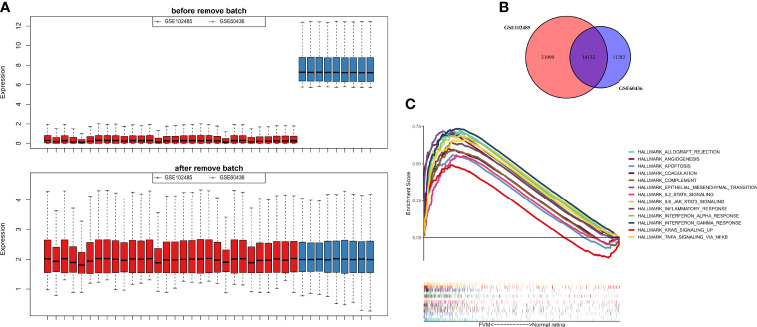
Data preprocessing and gene set enrichment analysis (GSEA). **(A)** Box plot for the expression profiles before and after normalization. **(B)** Venn diagram showing the intersection between expressed genes from two datasets. **(C)** GSEA plot for the significant Hallmark sets for FVM group (FDR<0.25, *p*<0.05).

The GSE60436 dataset used in this study is based on the platform of GPL6884 (Illumina HumanWG-6 v3.0 expression beadchip) and included 3 normal retina tissues and 6 FVM tissues. The second dataset used here, GSE102485, was analyzed on the GPL18573 Illumina NextSeq 500 platform and comprised 20 FVM samples(17 from type 2 diabetes mellitus patients and 3 from type 1 diabetes mellitus patients), 3 normal retina samples, and 7 other samples. It is clear from **Figure 2A** that the batch effects of the two different datasets were eliminated after internal correction and normalization. We finally selected 32 eligible samples (6 normal retinas and 26 FVMs) and 14,132 intersected genes from the two datasets for subsequent analysis (**Figure 2B**). Next, in the primary analysis of the biological differences between the two groups, GSEA revealed that several hallmarks of immune function were positively correlated with FVMs, including IL2-STAT5-signaling, IL6-JAK-STAT3-signaling, inflammatory response, IFN-α response, IFN-γ response, and TNF-α signaling *via* NF-κB. Other highly enriched hallmarks were angiogenesis, apoptosis, and epithelial mesenchymal transition, among others (**Figure 2C**). These results preliminarily indicate the correlation between immune activity and FVM in PDR.

### Immune Landscape of FVM and Normal Retina

**Figure 3:**
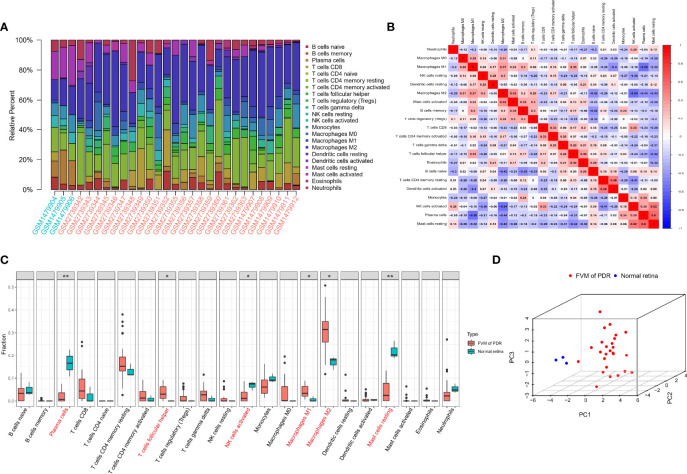
Immune infiltration landscape in FVM and normal retinal tissue. **(A)** Bar charts of 22 immune cell types in all eligible samples. **(B)** Correlations between infiltrating immune cells. Red and blue colors indicate positive and negative correlations, color intensity represents the degree of correlation. **(C)** Box plot of the difference in immune cell content between the FVM group and control group (*p < 0.05; **p < 0.01). **(D)** 3D scatter plot of PCA results.

The CIBERSORT algorithm was used to calculate the infiltration of 22 types of immune cells in all 32 samples (26 FVMs, and 6 normal retinas). The bar plot of **Figure 3A** shows the proportions of the 22 immune cell types in 29 eligible samples. **Figure 3B** indicates the correlations between immune cell types. The strongest positive correlation was between resting mast cells and plasma cells (r=0.6), while the strongest negative correlation was between plasma cells and M1 macrophages (r=-0.55). The differences in proportions of immune cell infiltration between the two groups is shown in **Figure 3C**. The percentages of follicular helper T cells, M1 macrophages, and M2 macrophages were increased significantly, while those of plasma cells, active NK cells and resting mast cells were decreased significantly compared with controls. Complete separation between the FVM and normal retina groups as shown in **Figure 3D** indicates that the infiltration of immune cell types may be used as a distinguishing characteristic of FVM in PDR patients.

### Identification of PDR-Infiltrating Immune Cell-Related Genes

**Figure 4:**
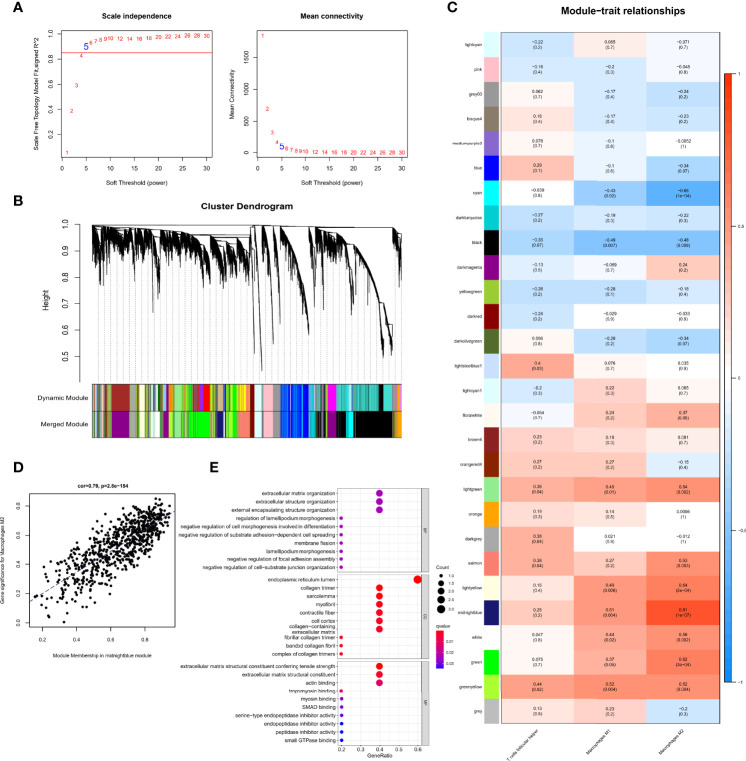
Identification of immune cell-related genes using WGCNA. **(A)** Analysis of the scale-free fit index for soft threshold powers (β=5). **(B)** Hierarchical dendrogram of the identified co-expression modules, indicated by color coding. **(C)** Heatmap plot of correlation between the gene module and immune cell type infiltration. Strength of the correlation is depicted by its color. **(D)** Correlations between the gene module memberships and gene significance for the midnight-blue module associated with M2 macrophages. **(E)** Bubble plots of GO enrichment analysis with hub genes in the midnight-blue module.

Gene expression data and immune cell types with significantly increase were used to conduct WGCNA, a powerful scale-free network used to identify co-expressed gene clusters correlated with meaningful immune cells based on the CIBERSORT algorithm, the CIBERSORT results of follicular helper T cells, M1 macrophages, and M2 macrophages in all 29 samples were selected as trait data for WGCNA. A scale-free topological network (soft-thresholding power=5, scale-free R2 = 0.9) was established (**Figure 4A**). Based on the hierarchical clustering method, genes that were highly co-expressed were clustered into different modules and color coded (**Figures 4B**). A total of 28 modules containing 14,132 genes were identified after merging modules with highly correlated eigengenes (**Figures 4B, C**). The ME, representing the expression of all the genes in this module, was associated with the characteristics of the three selected types of immune cells. Among them, we identified the midnight-blue module (r=0.81, p=1e-07) that was most closely associated with M2 macrophages (**Figures 4C, D**) as the hub module. The module contained 718 genes, of which 14 genes with kME>0.9 were considered hub genes and probably serve an essential pathobiological role (**Table S1**). GO enrichment analysis of the above 14 genes showed that several GO terms were enriched in tissue remodeling processes and fibrosis. The top 10 significant terms in the biological processes (BP), molecular functions (MF), and cellular components (CC) are shown in **Figure 4E**, including actin filament organization, extracellular matrix organization, myofibril and actin binding, among others. These provided additional evidence that these hub genes on M2 macrophages affect PDR progression.

### Exploration of M2 Macrophage-Related Biomarkers

**Figure 5:**
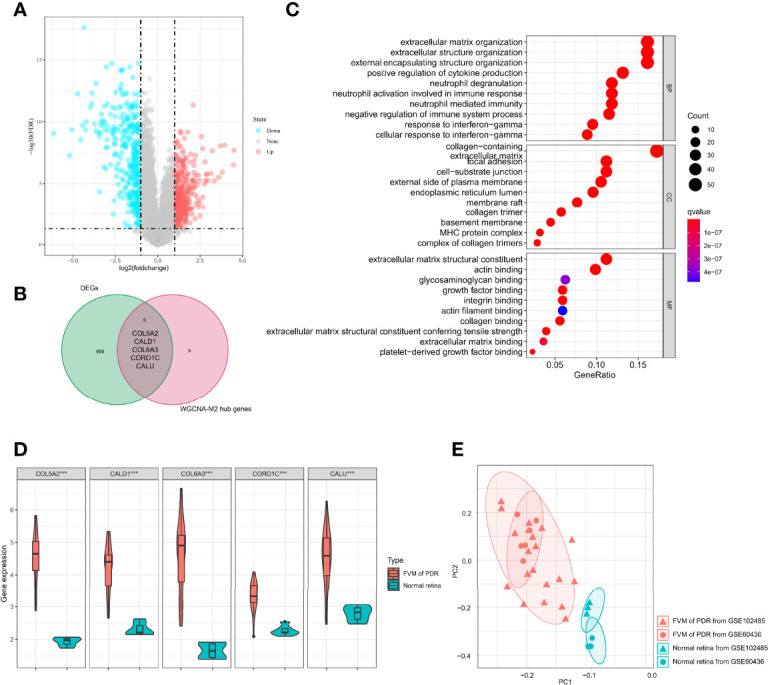
Identification of M2 macrophage-related biomarkers. **(A)** Volcano plot of DEGs between the FVM and control group. **(B)** Venn diagram of intersection genes between DEGs and M2 macrophage-related genes. **(C)** Bubble plots of GO enrichment analysis with five possible biomarkers. **(D)** Violin plot of expression levels of selected five genes in the FVM and normal retina group (***p<0.001). **(E)** PCA plot of 32 samples from two datasets (based on selected five genes, different shapes indicate different datasets, different colors indicate different sample types).

To identify possible genes that may contribute to the development of PDR, we analyzed gene expression data for all 32 samples and found 674 DEGs (**Table S2**, 328 upregulated and 346 downregulated) between FVM and normal retina (**Figure 5A**). A total of five genes (*COL5A2, CALD1, COL6A3, CORO1C*, and *CALU*) overlapped between DEGs and M2 macrophage-related genes (**Figure 5B**), and these five common genes constituted a proposed panel of possible biomarkers of PDR. GO functional enrichment analysis for these 5 genes showed that the top GO terms were also related to constructive processes like wound healing and tissue repair (**Figure 5C**), and the enrichment of these terms is consistent with the function of M2 macrophages. Furthermore, the expression of all five genes in the FVM group was significantly higher than that of controls (**Figure 5D**). PCA results showed complete separation between FVM samples and normal retinal samples both in the overall datasets and even in the individual dataset (**Figure 5E**).

## Discussion

In this study, we sought to identify the infiltration of immune cells in PDR. The CIBERSORT algorithm was used to calculate the infiltration of 22 types of immune cells in all 32 samples, including 26 FVMs and 6 normal retinas. We then identified the difference in the proportions of infiltrating immune cell types between the two groups. Compared to normal retinas, the percentages of follicular helper T cells, M1 macrophages, and M2 macrophages were increased significantly in FVMs. Using integrative analysis combining the differential expression and co-expression, we found that *COL5A2, CALD1, COL6A3, CORO1C*, and *CALU* are M2 macrophage-related biomarkers in PDR.

The retina is highly sensitive to the fluctuation of metabolic homeostasis owing to the combination of a limited vascular supply and high metabolic demand (25). The blood-retinal barrier not only protects the retina’s exposure but also regulates its supply of nutrients from the vasculature (26). Macrophages, astrocytes, and Müller cells play important cellular roles in the innate immunity of the blood-retinal barrier (27). PDR progression is closely associated with increasing abnormalities of the vasculature such as blood-retinal barrier dysfunction, neovascularization, and hemorrhage (28). However, few studies have focused on differences in composition of immune cells between PDR and normal retinal tissues. In this study, for the first time, we investigated the difference in proportions of infiltrating immune cell types between FVM and normal retina using CIBERSORT. We found that the percentages of follicular helper T cells, M1 macrophages, and M2 macrophages were increased significantly compared with controls. These findings provide new insights into the immune landscape of PDR based on bulk tissue transcriptomic profiling. However, the retina is a very complex neurosensory tissue with numerous types of cells (25), recent advances in single-cell RNA sequencing (scRNAseq) technology have provided more accurate results for characterizing different cell types in complex tissue (29). Due to the difficulty in obtaining normal retinal tissue and even complete PDR pathological samples, most of the existing single-cell based information has been established from experimental studies using diabetic animal models (30–32). In addition, currently available diabetic animal models mainly mimic non-proliferative diabetic retinopathy(NPDR) and rarely display retinal neovascularization, thereby limiting their use in PDR research (33). Accounting for the heterogeneity within normal and pathologic tissues, relevant mechanisms involved in different stages of the PDR process are worthy of further investigation according to samples with varying levels of DR severity. Future cell or spatial resolution transcriptome analysis are warranted to investigate the infiltration status of immune cell in the PDR to determine whether the regulation of immune status could be used as a potential therapeutic approach for PDR.

Macrophages are key regulators of tissue regeneration, repair, and fibrosis (34). The dysfunctional macrophage can lead to uncontrolled generation of growth factors, impaired production of anti-inflammatory macrophages, or erroneous interactions between macrophages and endothelial cells, epithelial cells, fibroblasts, and tissue or stem progenitor cells (35). Aberrant repair contributes to sustained damage, and this could induce the progression of pathological fibrosis (36, 37). A recent study has reported that M2 macrophages participate actively in the natural process of oxygen−induced retinopathy(OIR) in mice by affecting pathological neovascularization and fibrosis (38). Another single-cell transcriptome study also showed different fibrotic, gliotic, and inflammatory profiles in microglia/macrophages subpopulations with effects on the severity of diabetic retinopathy in the Akimba mouse model (54). Which were in accordance with our results from GO enriched analysis of 14 hub genes and 5 potential biomarkers related to M2 macrophages. Meanwhile, Clinical research has also provided relevant evidence. One study used optical coherence tomography angiography (OCTA) to report that the density of macrophage-like cells was significantly increased in PDR (39). Similarly, another study suggested that the hyperreflective spots visualized by optical coherence tomography (OCT) might be related to the presence of activated microglia/macrophage in diabetic macular edema (40).

In this study, we revealed that the *COL5A2, CALD1, COL6A3, CORO1C*, and *CALU* genes associated with M2 macrophages and showed high expression in PDR by differential expression and co-expression analyses. A recent comparative analysis of the transcriptome of FVM of PDR and inner limiting membrane (ILM) of non-diabetic epiretinal membranes has shown that the signature of FVM is characterized by the expression of a variety of extracellular matrix (ECM) proteins and several collagen types, as well as the accumulation of endothelial cells, M2 macrophages and myofibroblasts (41). These results are in accordance with our findings of bioinformatics analysis. The collagen type V alpha 2 (*COL5A2*) gene offers a template for a component of type V collagen and participates in the formation of pathological scarring (42), previous studies have reported a positive association between expression of *COL5A2* and macrophages as well as proliferation, malignant transformation, and resistance to apoptosis in a variety of tumors (43). We report for the first time that *COL5A2* gene is highly expressed in PDR and associated with M2 macrophages. Further studies with a larger clinical cohort and basic research will be needed to further confirm and extend these findings. Caldesmon 1 (*CALD1*) performs as a cytoskeleton-associated protein and regulates cell morphology and motility *via* actin filament modulation. It plays roles in cytoskeletal organization, cell adhesion, and vascularization (44). Previously, it has been shown that *CALD1* may stimulate and polarize tumor-associated macrophages (45), and this is consistent with our findings but the specific mechanism remains poorly understood and further investigations are needed. Collagen Type VI Alpha 3 (*COL6A3*) is associated with insulin resistance and adipose tissue inflammation (46). Previous reports have supported a role for *COL6A3* in inflammation obesity and obesity-associated insulin resistance which may lead to a higher incidence and more rapid progression of diabetes complications (47, 48). In the current study, we report for the first time that *COL6A3* is highly expressed in FVMs and is associated with M2 macrophage cells in PDR. Further research will be necessary to gain insight into such potential mechanisms. Coronin-like actin-binding protein 1C (*CORO1C*) belongs to the coronin family of actin-binding proteins that are important for the control and remodeling of the actin filaments network (49). One report has indicated that the PI3K/AKT signaling pathway is significantly inhibited by *CORO1C* knockdown in colorectal cancer (50). The expression of *CORO1C* in PDR has not been previously reported. In this study, we found that *CORO1C* is associated with M2 macrophage cells in PDR. Further mechanistic studies are required to understand the relevant mechanism of action. Calumenin (*CALU*) is a multiple EF-hand Ca2+-binding protein (51). One report has indicated a positive correlation of *CALU* with cancer-associated fibroblasts and macrophages in bladder cancer (52). Moreover, previous analyses of *CALU* target genes were mainly focused on epithelial-mesenchymal transition-related genes (53). In this study, we found the association between CALU and M2 macrophage cells in PDR. However, the mechanisms by which CALU regulates are not clear. Combining the obtained biomarkers and the aforementioned PDR immune landscape results, immunomodulation or targeted therapy for specific therapeutic biomarkers may be potential therapeutic approaches to inhibit FVM formation and slow or even reverse the progression of PDR.

## Conclusion

Taken together, using integrative analysis combining differential expression and co-expression, we found that the ratio of follicular helper T cells, M1 macrophages, and M2 macrophages increased significantly in PDR, while the ratio of plasma cells, activated NK cells and resting mast cells was decreased compared with controls. These findings indicate that M2 macrophages play an important role in the progression of PDR, and further study of *COL5A2, CALD1, COL6A3, CORO1C*, and *CALU* could inform novel ideas and basis for the understanding of disease progression and targeted treatment of PDR.

## Data Availability Statement

The original contributions presented in the study are included in the article/**Supplementary Material**. Further inquiries can be directed to the corresponding author.

## Author Contributions

Conceptualization: JL. Methodology and software: ZM, YC, and WW. Visualization: ZM and XY. Formal analysis and data curation: BY, YM, YL, and XY. Writing: ZM and YC. Review and editing: WW and JL. Supervision and acquisition of funding: JL. All authors have read and agreed to the submitted version of the manuscript.

## Funding

This work was supported by grants from the National Natural Science Foundation of China (81570847) and the Hunan Provincial Natural Science Foundation of China (2020JJ4800).

## Conflict of Interest

The authors declare that the research was conducted in the absence of any commercial or financial relationships that could be construed as a potential conflict of interest.

## Publisher’s Note

All claims expressed in this article are solely those of the authors and do not necessarily represent those of their affiliated organizations, or those of the publisher, the editors and the reviewers. Any product that may be evaluated in this article, or claim that may be made by its manufacturer, is not guaranteed or endorsed by the publisher.
